# Supplementary material for: Exploring temperature and humidity environment combined with air quality index, black carbon, the short-term effect of combined exposure on respiratory disease mortality in Southwest China
Source: PLoS One. 2025 Apr 18;20(4):e0319545. doi: 10.1371/journal.pone.0319545 (PMC12007920; doi:10.1371/journal.pone.0319545)
Supplement: S1 File — Table S1. Relative risk of combined events T1A1 T1A2 T1A3. Table S2. Relative risk of combined events T2A1 T2A2 T2A3. Table S3. Relative risk of combined events T3A1 T3A2 T3A3. Table S4. Relative risk of combined events T1B1 T1B2 T1B3. Table S5. Relative risk of combined events T2B1 T2B2 T2B3. Table S6. Relative risk of combined events T3B1 T3B2 T3B3. Table S7. The results of AIC(BIC) of combined events definition. Table S8. Sensitivity analysis for cumulative effect estimates of combined event (T3A1,T3B1) on respiratory mortality at lag 0 and lag 0-7 days. Table S9. Sensitivity analysis for cumulative effect estimates of combined event (T3A2,T3B2) on respiratory mortality at lag 0 and lag 0-7 days. (DOCX) [file pone.0319545.s001.docx]

**Exploring Temperature and Humidity Environment Combined with Air Quality Index,Black carbon,the short-term Effect of Combined Exposure on Respiratory Disease Mortality in Southwest China**

Hengyu Su^1¶^ Di Wu^1&^ Song Chen^2^ KaiYang Guo^3^ Huifang Xie^1*^

1.School of Public Health, Xinjiang Medical University, Urumqi, Xinjiang, China

2.School of Health Management, Xinjiang Medical University, Urumqi, Xinjiang, China

3.school of nursing,Xinjiang Medical University, Urumqi, Xinjiang, China

*Corresponding auther：Huifang Xie

E-mail：xhfworld@sina.com

**Hengyu Su**

**E-mail：**[526999839@qq.com](mailto:526999839@qq.com)

**Huifang Xie**

**E-mail：**[xhfworld@sina.com](mailto:xhfworld@sina.com)

Table.S1 Relative risk of combined events T1A1 T1A2 T1A3..............................................................3

Table.S2 Relative risk of combined events T2A1 T2A2 T2A3..............................................................3

Table.S3 Relative risk of combined events T3A1 T3A2 T3A3..............................................................3

Table.S4 Relative risk of combined events T1B1 T1B2 T1B3..............................................................4

Table.S5 Relative risk of combined events T2B1 T2B2 T2B3..............................................................4

Table.S6 Relative risk of combined events T3B1 T3B2 T3B3..............................................................4

Table.S7 The results of AIC(BIC) of combined events definition.........................................................5

Table.S8 Sensitivity analysis for cumulative effect estimates of combined event (T3A1,T3B1) on respiratory mortality at lag 0 and lag 0-7 days......................................................................................5

Table.S9 Sensitivity analysis for cumulative effect estimates of combined event (T3A2,T3B2) on respiratory mortality at lag 0 and lag 0-7 days......................................................................................6

Table.S1 Relative risk of combined events T1A1 T1A2 T1A3

|  | TIA1 | T1A2 | T1A3 |
| --- | --- | --- | --- |
|  | RR1(95%*CI*) | RR2(95%*CI*) | RR3(95%*CI*) |
| lag0 | 1.07(0.98,1.16) | 1.09(0.99,1.19) | 1.14(0.99,1.29) |
| lag1 | 1.08(0.98,1.18) | 1.10(1.00,1.20) | 1.16(1.02,1.30) |
| lag2 | 1.11(1.01,1.22) | 1.11(1.01,1.22) | 1.20(1.08,1.33) |
| lag3 | 1.09(1.00,1.19) | 1.13(1.04,1.22) | 1.26(1.11,1.41) |
| lag4 | 1.08(0.99,1.17) | 1.12(1.01,1.23) | 1.23(1.09,1.37) |
| lag5 | 1.07(0.97,1.17) | 1.10(0.99,1.21) | 1.19(1.06,1.32) |
| lag6 | 1.05(0.96,1.15) | 1.09(0.98,1.20) | 1.15(1.02,1.28) |
| lag7 | 1.04(0.94,1.14) | 1.08(0.96,1.19) | 1.10(0.98,1.22) |

Table.S2 Relative risk of combined events T2A1 T2A2 T2A3

|  | T2A1 | T2A2 | T2A3 |
| --- | --- | --- | --- |
|  | RR1(95%*CI*) | RR2(95%*CI*) | RR3(95%*CI*) |
| lag0 | 0.79(0.65,0.93) | 0.99(0.93,1.05) | 1.10(0.99,1.21) |
| lag1 | 0.82(0.66,0.99) | 1.01(0.95,1.07) | 1.11(1.01,1.22) |
| lag2 | 0.89(0.70,1.08) | 1.04(0.97,1.11) | 1.15(1.04,1.26) |
| lag3 | 0.97(0.79,1.15) | 1.06(0.98,1.14) | 1.12(0.99,1.25) |
| lag4 | 0.95(0.77,1.13) | 1.05(0.97,1.13) | 1.09(0.98,1.20) |
| lag5 | 0.91(0.73,1.09) | 1.03(0.96,1.10) | 1.08(0.96,1.20) |
| lag6 | 0.89(0.72,1.06) | 1.02(0.95,1.09) | 1.05(0.95,1.15) |
| lag7 | 0.88(0.70,1.06) | 1.01(0.93,1.09) | 1.03(0.92,1.14) |

Table.S3 Relative risk of combined events T3A1 T3A2 T3A3

|  | T3A1 | T3A2 | T3A3 |
| --- | --- | --- | --- |
|  | RR1(95%*CI*) | RR2(95%*CI*) | RR3(95%*CI*) |
| lag0 | 1.07(0.96,1.18) | 1.14(0.98,1.30) | 1.14(1.04,1.24) |
| lag1 | 1.10(1.01,1.19) | 1.17(1.00,1.34) | 1.29(1.15,1.43) |
| lag2 | 1.17(1.07,1.27) | 1.18(1.02,1.35) | 1.43(1.16,1.69) |
| lag3 | 1.15(1.05,1.25) | 1.19(1.03,1.36) | 1.55(1.20,1.91) |
| lag4 | 1.07(0.94,1.20) | 1.19(1.02,1.37) | 1.46(1.17,1.75) |
| lag5 | 1.05(0.92,1.18) | 1.18(1.01,1.35) | 1.40(1.07,1.73) |
| lag6 | 1.04(0.92,1.16) | 1.17(1.00,1.34) | 1.36(0.99,1.73) |
| lag7 | 1.03(0.91,1.15) | 1.15(0.99,1.31) | 1.34(0.98,1.70) |

Table.S4 Relative risk of combined events T1B1 T1B2 T1B3

|  | T1B1 | T1B2 | T1B3 |
| --- | --- | --- | --- |
|  | RR1(95%*CI*) | RR2(95%*CI*) | RR3(95%*CI*) |
| lag0 | 1.02(0.84,1.20) | 1.07(0.95,1.20) | 1.18(1.04,1.33) |
| lag1 | 1.01(0.84,1.18) | 1.06(0.92,1.20) | 1.16(1.03,1.29) |
| lag2 | 0.98(0.78,1.18) | 1.04(0.91,1.17) | 1.14(1.01,1.27) |
| lag3 | 0.97(0.79,1.15) | 1.00(0.89,1.11) | 1.12(0.99,1.25) |
| lag4 | 0.93(0.78,1.08) | 0.99(0.85,1.13) | 1.10(0.97,1.23) |
| lag5 | 0.89(0.73,1.05) | 0.96(0.85,1.07) | 1.08(0.95,1.21) |
| lag6 | 0.84(0.67,1.01) | 0.96(0.84,1.08) | 1.06(0.94,1.18) |
| lag7 | 0.75(0.50,1.00) | 0.89(0.76,1.02) | 1.05(0.93,1.17) |

Table.S5 Relative risk of combined events T2B1 T2B2 T2B3

|  | T2B1 | T2B2 | T2B3 |
| --- | --- | --- | --- |
|  | RR1(95%*CI*) | RR2(95%*CI*) | RR3(95%*CI*) |
| lag0 | 1.17(1.02,1.33) | 1.18(1.04,1.32) | 1.28(1.12,1.45) |
| lag1 | 1.16(1.01,1.31) | 1.17(1.03,1.31) | 1.27(1.10,1.44) |
| lag2 | 1.13(0.98,1.28) | 1.14(1.02,1.26) | 1.22(1.09,1.35) |
| lag3 | 1.10(0.96,1.24) | 1.09(1.01,1.17) | 1.18(1.02,1.34) |
| lag4 | 1.07(0.98,1.17) | 1.05(1.00,1.10) | 1.15(0.99,1.31) |
| lag5 | 0.97(0.90,1.04) | 1.03(0.97,1.09) | 1.13(0.98,1.28) |
| lag6 | 0.98(0.89,1.07) | 0.98(0.93,1.03) | 1.08(0.96,1.20) |
| lag7 | 0.98(0.88,1.08) | 0.97(0.88,1.06) | 1.06(0.94,1.18) |

Table.S6 Relative risk of combined events T3B1 T3B2 T3B3

|  | T3B1 | T3B2 | T3B3 |
| --- | --- | --- | --- |
|  | RR1(95%*CI*) | RR2(95%*CI*) | RR3(95%*CI*) |
| lag0 | 1.34(1.16,1.52) | 1.87(1.21,2.53) | - |
| lag1 | 1.22(1.11,1.34) | 1.69(1.16,2.23) | - |
| lag2 | 1.17(1.04,1.31) | 1.43(1.13,1.73) | - |
| lag3 | 1.14(1.00,1.29) | 1.28(1.09,1.47) | - |
| lag4 | 1.10(0.98,1.23) | 1.09(1.00,1.19) | - |
| lag5 | 1.05(0.97,1.13) | 1.07(0.98,1.17) | - |
| lag6 | 1.02(0.95,1.09) | 1.04(0.97,1.11) | - |
| lag7 | 0.97(0.89,1.05) | 0.98(0.91,1.05) | - |

Table.S7 The results of AIC(BIC) of combined events definition

| THI | | | BC | | | AQI | | |
| --- | --- | --- | --- | --- | --- | --- | --- | --- |
| AIC | BIC | dot | AIC | BIC | dot | AIC | BIC | dot |
| 11354.36 | 11381.91 | 3 | 11437.41 | 11459.45 | 3 | 13620.63 | 13671.86 | 3 |
| 11355.20 | 11388.26 | 4 | 11439.33 | 11466.88 | 4 | 13612.99 | 13669.91 | 4 |
| 11357.27 | 11395.84 | 5 | 11441.03 | 00474.09 | 5 | 13614.42 | 13677.03 | 5 |

Table.S8 Sensitivity analysis for cumulative effect estimates of combined event (T3A1,T3B1) on respiratory mortality at lag 0 and lag 0-7 days

| Model | NS(X) | （df=） | Lag0（RR,95%*CI*） | Lag07（RR,95%*CI*） |
| --- | --- | --- | --- | --- |
| T3A1 | O_38h_ | 3 | 1.07(0.96,1.18) | 2.22(1.48,2.96) |
|  |  | 5 | 1.07(0.96,1.18) | 2.22(1.48,2.96) |
|  |  | 6 | 1.07(0.97,1.18) | 2.22(1.48,2.96) |
|  | AP | 3 | 1.07(0.96,1.18) | 2.22(1.48,2.96) |
|  |  | 5 | 1.07(0.96,1.18) | 2.22(1.48,2.96) |
|  |  | 6 | 1.07(0.96,1.18) | 2.22(1.48,2.96) |
| T3B1 | O_38h_ | 3 | 1.34(1.16,1.52) | 1.97(0.96,2.96) |
|  |  | 5 | 1.35(1.17,1.52) | 1.97(0.96,2.97) |
|  |  | 6 | 1.34(1.17,1.52) | 1.97(0.96,2.96) |
|  | AP | 3 | 1.34(1.16,1.52) | 1.97(0.96,2.96) |
|  |  | 5 | 1.34(1.16,1.52) | 1.97(0.96,2.96) |
|  |  | 6 | 1.34(1.16,1.52) | 1.97(0.96,2.96) |
|  | PM_2.5_ | 3 | 1.34(1.16,1.52) | 1.97(0.96,2.96) |
|  |  | 5 | 1.34(1.16,1.52) | 1.97(0.96,2.96) |
|  |  | 6 | 1.34(1.16,1.52) | 1.97(0.96,2.97) |
|  | PM_10_ | 3 | 1.34(1.16,1.52) | 1.97(0.96,2.96) |
|  |  | 5 | 1.34(1.18,1.50) | 1.97(0.96,2.97) |
|  |  | 6 | 1.34(1.17,1.50) | 1.97(0.97,2.97) |
|  | CO | 3 | 1.34(1.16,1.52) | 1.97(0.96,2.96) |
|  |  | 5 | 1.34(1.16,1.52) | 1.97(0.96,2.96) |
|  |  | 6 | 1.34(1.16,1.52) | 1.97(0.96,2.96) |
|  | NO_2_ | 3 | 1.34(1.16,1.52) | 1.97(0.96,2.96) |
|  |  | 5 | 1.34(1.16,1.52) | 1.97(0.96,2.96) |
|  |  | 6 | 1.34(1.17,1.52) | 1.97(0.96,2.97) |

Table.S9 Sensitivity analysis for cumulative effect estimates of combined event (T3A2,T3B2) on respiratory mortality at lag 0 and lag 0-7 days

| Model | Lag0（RR,95%*CI*） | Lag07（RR,95%*CI*） |
| --- | --- | --- |
| T3A2+PM_2.5_ | 1.15(0.98,1.32) | 3.12(1.76,4.48) |
| T3A2+PM_2.5_+CO | 1.16(0.96,1.36) | 3.13(1.77,4.49) |
| T3A2+PM_2.5_+CO+PM_10_ | 1.14(0.97,1.31) | 3.13(1.76,4.50) |
| T3A2+PM_2.5_+CO+PM_10_+SO_2_ | 1.14(0.98,1.30) | 3.12(1.76,4.47) |
| T3A2+PM_2.5_+CO+PM_10_+SO_2_+NO_2_ | 1.14(0.98,1.30) | 3.12(1.76,4.48) |
| T3B2+SO_2_ | 1.19(1.05,1.33) | 2.24(1.55,2.93) |
| T3B2+Wind | 1.18(1.04,1.31) | 2.23(1.55,2.91) |
| T3B2+SO_2_+Wind | 1.18(1.04,1.32) | 2.23(1.55,2.91) |
