# Supplementary material for: Exploring temperature and humidity environment combined with air quality index, black carbon, the short-term effect of combined exposure on respiratory disease mortality in Southwest China
Source: PLoS One. 2025 Apr 18;20(4):e0319545. doi: 10.1371/journal.pone.0319545 (PMC12007920; doi:10.1371/journal.pone.0319545)
Supplement: Attachment — Submitted filename: Response to Reviewers1.docx [file pone.0319545.s002.docx]

**List of Responses:**

Dear Dr.academic editor and reviewers:

Thank you for your letter and for the reviewers’ comments concerning our manuscript

entitled “Exploring Health Effects under Specific Causes of Respiratory Mortality Based on

Different Definitions of Air Quality Index,Black carbon,Temperature and Humidity environment Combined Exposure in Southwest,China” (ID:PONE-D-24-49828 ). Those comments are all valuable and very helpful for revising and improving our paper, as well as the important guiding significance to our researches. We have studied comments carefully and have made correction which we hope meet with approval. Revised portion are marked in red in the paper.

In your letter, we noticed that your journal stated, "When completing the data availability statement of the submission form, you indicated that you will make your data available on acceptance. " However, upon reviewing our submission, we found that we indicated, "No - some restrictions will apply," and added, "This text is appropriate if the data are owned by a third party and authors do not have permission to share the data".

We appreciate your attention to and review of our paper. In response to your request for the upload of raw data, we acknowledge the significance of this requirement. However, due to the sensitive nature of our raw data, we are unable to share it publicly. We can, however, provide the necessary data summaries and analysis results that support our research conclusions. If there are alternative solutions or options you would like to discuss, please let us know, and we will do our best to accommodate.

Thank you for your understanding and support!

Best wish！
HengYu Su
2025/1/2

**The main corrections in the paper and the responds to the reviewer’s comments are as flowing:**

**Responds to the reviewer’s comments:**

**Academic editor：**

1.The major negative aspects of the paper include the lack of novelty in methodology, as the study relies heavily on existing models like DLNM and GAM without substantial innovation or advancement in approach.

2.The paper’s regional focus and reliance on single-area data limit its generalizability to other climate zones or socioeconomic contexts. The findings, while region-specific, do not significantly differ from prior research, diminishing their broader applicability. The paper overemphasizes known relationships, such as the ‘U’-shaped THI curve, without exploring the underlying mechanisms in depth.

3.The focus on combined exposure events (THI, AQI, and BC) lacks sufficient clarity in establishing unique causal pathways, which limits the study's ability to provide actionable recommendations.

4.Additionally, the definitions of combined events (T1, T2, T3) appear arbitrary and lack robust justification, raising concerns about the consistency and validity of results.

5.The reliance on lag effects up to 3 days, though discussed extensively, does not sufficiently explore longer-term impacts, which could provide a more comprehensive understanding of respiratory mortality.

6.The study's conclusions reiterate known risks without presenting significant new insights, while the suggestions for health early warning systems remain vague and impractical for implementation.

**Response to academic editor：**

**Thank you for your insightful comments. Your feedback is both constructive and practical, significantly contributing to the enhancement of the article's quality. We will address each of your comments and suggestions in our revisions. Our responses are as follows:**

**1.**Thank you very much for your evaluation and suggestions. Prior to conducting our research, our team analyzed a substantial number of similar articles and consulted experts in relevant fields. In conjunction with our own research, we determined that the two models, DLNM and GAM, are the most suitable methods for our study. Currently, we are acquainted with emerging methodologies. For instance, the Bayesian Kernel Machine Regression Model (BKMR) has been developed in recent years for the analysis of pollutants. However, this model is not yet fully matured. Notably, it is primarily focused on correlation analysis between multiple pollutants, which does not align with our research methods. Additionally, the recently popular spatiotemporal models for pollutants are also inconsistent with our research data and methodologies. Consequently, the two models, DLNM and GAM, are the most appropriate methods for our research data and objectives. Notably, both methods are well-established and exhibit minimal shortcomings. We first employ the GAM to analyze nonlinear relationships in time series data, fitting potential patterns and trends using various smoothing functions. Subsequently, we utilize the DLNM to capture the dynamic changes in both time and dose effects, effectively addressing the complex relationships between environmental factors and health outcomes. Consequently, our data analysis is both effective and scientifically rigorous. Moreover, the majority of pertinent research in the field relies solely on one model for comprehensive analyses. In contrast, we integrate both models in our analysis, thereby leveraging their respective advantages. Thus, our analytical approach is both novel and practical. Based on our analysis, we will also elaborate on the benefits of integrating the two models in the methods section to enhance both the novelty and applicability of our approach. The supplementary content is as follows: Initially, we employ the GAM to analyze nonlinear relationships within time series data, effectively fitting potential patterns and trends using various smoothing functions. Subsequently, we utilize the DLNM to dynamically capture the temporal and dose-response effects, addressing the intricate relationships between environmental factors and health outcomes. This approach culminates in the establishment of an exposure-lag-response model. When used in conjunction, the two can fully leverage their complementary characteristics, thereby enhancing the novelty, depth, and breadth of the analysis.

**2.**Thanks very much to the expert for this advice.

①While this suggestion is valuable, it also represents a limitation of our study, specifically regarding regional applicability. The results of our research and the models developed for the early warning systems cannot be directly extrapolated to regions in different climate zones. This limitation arises from the fact that our study was conducted in an area characterized by consistently high temperatures and humidity throughout the year, conditions that may not be present in other climate zones. The primary aim of our research is to analyze the impact of combined exposure events to THI, AQI, and BC on respiratory mortality within the population. The methodologies, strategies, and findings of our study can serve as a reference for other regions, which is also a significant objective of our research. Additionally, we have incorporated a discussion on the limitations of our study, informed by expert opinions, as follows: To enhance and broaden the findings of this study, it is essential to incorporate additional research conducted in diverse climate zones. Additional relevant studies are necessary to enhance the reliability of these findings.

②Before conducting this study, we performed an extensive literature review and in-depth exploration of works in related fields. Unfortunately, we did not identify any relevant literature reports that utilized a combination of THI, AQI, and BC to investigate their interactions and establish an early warning system. Our study focuses on a specific area, and the results are derived from the distinct research, analysis, and discussion of three types of factors. However, based on the rigorous scientific data and analysis presented in this article, which aligns with the findings of most studies, the general outcome suggests a broader applicability. But we also mentioned in the article discussion section:Notably, the THI threshold results diverge from the conventional grading definitions. The average concentration and variation range of BC observed in this study were lower than those reported in previous studies. And explained the reason: This discrepancy may be attributed to China's vigorous efforts to control air pollution in recent years. However ,we have incorporated comparable research findings from other regions in alignment with your insights, as detailed below: Liang also found that, compared with high temperature conditions, hot and humid weather in Shanghai was significantly associated with a higher risk of outpatient visits and an increased risk of all-cause morbidity^15^. However, these findings are inconsistent with the research results of Fallah Ghalhari G, who found that the heat stress excitation threshold is inversely related to mortality^29^.This discrepancy may be attributed to the lower humidity levels in the Middle East, as well as variations in socioeconomic factors among the study samples. The primary focus of our article is to examine the impact of combined exposure events of THI, AQI, and BC on respiratory mortality outcomes. To our knowledge, there are limited studies addressing the combined effects of these three factors. Therefore, in this article, we present the results of all combined exposure events, explore potential influencing factors and causes, and discuss prospects for future research in the Discussion section.

③The impact of the THI on the population exhibits a 'U' shaped relationship, as THI is derived from a combination of daily average temperature and relative humidity. One notable characteristic of this relationship is the existence of an optimal interval. This finding aligns with the predominant results in scientific research and is consistent with reports on THI from other regions, thereby reinforcing the validity of our data and the scientific rigor of our analysis. Additionally, we have incorporated relevant supplements regarding the mechanism of action of THI based on your feedback, as detailed below: Although the biological mechanisms underlying thermohumidity events remain unclear, one possible explanation is that during low THI events, reduced humidity impairs human respiratory mucosal function and ciliary motility. This impairment facilitates the adherence of bacteria to the mucosa, thereby increasing the risk of infection^30^. An experiment demonstrates that mice exposed to low humidity exhibit impaired mucociliary clearance, diminished innate antiviral defenses, and compromised tissue repair, resulting in increased susceptibility to inflammasome caspase-mediated diseases^31^. During high THI events, elevated temperatures combined with high humidity can hinder the body's ability to dissipate heat. This may result in heat-related illnesses such as heat stroke and heat cramps, while also increasing the strain on the human respiratory system^32,33^

30.Liener K, Leiacker R, Lindemann J, et al. Nasal mucosal temperature after exposure to cold, dry air and hot, humid air. *Acta Otolaryngol*. 2003 Sep;123(7):851-6. doi: 10.1080/00016480310000601a

31.Kudo E, Song E, Yockey LJ, et al. Low ambient humidity impairs barrier function and innate resistance against influenza infection. *Proc Natl Acad Sci U S A*. 2019 May 28;116(22):10905-10910. doi: 10.1073/pnas.1902840116.

32.Fouillet A, Rey G, Laurent F,et al. Excess mortality related to the August 2003 heat wave in France. *Int Arch Occup Environ Health*. 2006 Oct;80(1):16-24. doi: 10.1007/s00420-006-0089-4.

33. Kenny GP, Wilson TE, Flouris AD, et al. Heat exhaustion. Handb Clin Neurol. 2018;157:505-529. doi: 10.1016/B978-0-444-64074-1.00031-8.

**3.**Thank you for your valuable views, questions, and comments. Firstly, based on your feedback, our team re-evaluated the entire experimental design and process. This included reorganizing the data, selecting the optimal method according to the characteristics and type of data, and conducting scientific and rigorous modeling calculations, which yielded consistent results. Consequently, we affirm that our research is accurate, and the causal relationships are overall clear and unambiguous. Secondly, we conducted an extensive review of the literature and found that the results of our research methods align with those of other similar studies. Lastly, we meticulously reviewed the discussion section and identified certain logical inconsistencies that could lead to confusion regarding cause and effect. We sincerely appreciate your insights. In response to your and other reviewers' comments, we have thoroughly supplemented each modeling and calculation step in our research design to ensure that the causal pathways are clearly articulated. The supplementary content is as follows: We meticulously cleaned, screened, and organized the data in alignment with the objectives of the research. Initially, specific time periods and diseases were identified, and data were excluded for individuals who were not present in the area during their lifetime, as well as for those who resided in the area for less than five years prior to their death. We subsequently removed any missing values and cross-verified values that deviated by more than three standard deviations from the long-term mean. Based on your feedback, we have revised certain statements in the discussion section that contained confusing expressions and reversed logic. The modified sections are highlighted in red within the article.

**4.**We appreciate your valuable feedback. This oversight in our writing has been addressed. In response to your suggestions, we have provided a more detailed explanation of the definitions and underlying reasons in the statistical methods section, as follows: The definition of exposure events is informed by the RCS exposure response curve, in conjunction with the 'GB3095-2012' standard established by the World Meteorological Organization (WMO), the National Meteorological Administration, and the China Meteorological Administration for heat wave definitions, as well as previous research. We categorize the Temperature-Humidity Index (THI) as follows: T1 (THI < 63), T2 (63 ≤ THI < 75), and T3 (THI ≥ 75). Furthermore, by integrating the BC and AQI percentiles from this study and referencing the Technical Specifications for Ambient Air Quality Assessment (HJ6632013), the Guidelines for the Preparation of Urban Heavy Air Pollution Emergency Plans, and the Sichuan Province Heavy Pollution Weather Emergency Plan (2018 Revision) - Quantitative Indicators, we redefine the BC and AQI categories as B1 (P≥50), B2 (P≥75), B3 (P≥90), A1 (P≥50), A2 (P≥75), and A3 (P≥90). Notably, A1 encompasses A2 and A3, while B1 includes B2 and B3. This study utilizes the RCS intersection value as a node, treating the combined meteorological event variable as a continuous variable, thereby fixing it within the ranges above or below different percentiles. This approach aims to investigate the relationship between THI and extreme AQI and BC, as well as to explore the impact of exposure to combined events with varying threshold ranges on respiratory mortality, ultimately refining the thresholds for early warning models.

**5.** Thank you very much for your professional review. Our team also evaluated the necessity of long-term exploration at the outset of the research. Ultimately, however, we decided against pursuing long-term exploration in this article based on the study's objectives. Firstly, the meaningful results derived from long-term exploration span an extensive timeframe, which does not align with our research goals or the requirement for establishing an early warning system. Additionally, our investigation into short-term effects has yielded significant and practical results, accompanied by a rich discussion that sufficiently supports the design and objectives of our study. Finally, in the context of establishing an early warning system, short-term effects are most relevant. Long-term effects necessitate prolonged follow-up of the exposed population, which does not correspond with the aims of our study. Therefore, we opted not to explore long-term effects in this paper. However, in response to your suggestions, we have revised the title and limitations to more effectively present the short-term effects. The modifications are as follows: **Exploring Temperature and Humidity Environment Combined with Air Quality Index,Black carbon,the short-term Effect of Combined Exposure on Respiratory Disease Mortality in Southwest China.**

Additionally, this study primarily concentrated on short-term effects and did not fully investigate the long-term outcomes related to combined exposure events and respiratory deaths. This limitation may affect the comprehensiveness of the findings. To enhance and broaden the findings of this study, it is essential to incorporate additional research conducted in diverse climate zones, additional relevant studies are necessary to enhance the reliability of these findings.

**6**.We appreciate your insightful feedback. Our findings regarding individual exposure events align with previous research, underscoring the significance of our study. Given the limited literature on combined exposure events involving THI, AQI, and BC, much of the discourse presented in this article represents novel research contributions. We acknowledge the reviewers' concerns regarding the insufficient discussion of the early warning system, which we also overlooked during the writing process. In response to your valuable suggestions, we have made pertinent additions and revisions to the discussion section, detailed as follows: In light of the potential exposure lag response curve, cumulative relative risk results, and the effectiveness of risk early warning systems, we have thoroughly considered and discussed the establishment of an early warning system. Firstly, we recommend that the local climate environment, as well as economic and social conditions, be comprehensively evaluated. The thresholds for various influencing factors can vary significantly depending on the climate zone, and economic conditions may also influence the quality and standards of the early warning system. Secondly, given the hysteresis associated with the impacts, we propose extending the setting time for the early warning system and issuing alerts within one week following the occurrence of comprehensive exposure events to enhance population protection and prevention efforts. Furthermore, for the specific development and dissemination of the early warning system, we suggest collaboration with relevant agencies, such as the local CDC. This partnership should leverage the expertise of the CDC’s infectious disease and environmental institutes, ensuring mutual support in providing technical assistance. Subsequently, relevant outreach departments should promote the system through various media channels to ensure that the majority of residents understand, recognize, and accept this system, thereby enhancing its practical implementation. Lastly, we recommend categorizing heat wave warning levels for exposure events into three tiers: low (T2), medium (T1), and high (T3). Given the heightened risks associated with combined events, we further propose dividing pollutant warnings into three categories based on the definitions of AQI and BC concentrations: '+Low', '+Medium', and '+High'.

**Reviewer#1：**

**This article presents a novel perspective on a relevant topic, closely aligning with current research hotspots. It offers a comprehensive, scientific, and detailed analysis, accompanied by an in-depth discussion and practical significance. Furthermore, it proposes feasible measures. Overall, this work meaningful. However, there remain several areas for improvement in the current version of the paper. More precisely.**
**1. In the "Main Content" section, the formula for model construction, "Y = β0+β1X1+β2X2+ ... + βnXn+ε", requires improved spacing for clarity. It should be formatted as "Y = β0 + β1X1 + β2X2 + ... + βnXn + ε".**

**Response:**Thank you very much for your careful observations and advice. We've added the interval to the formula.

**2. In the main section, the model builds, "Since the dependent and independent variables are continuous, we use the ordinary least squares regression model ols () function to build the model:" the colon should be a period to correctly end the sentence.**

**Response:**Thanks very much for your comments. We've changed it to the correct colon .

**3. In section MAIN CONTENT, the spacing around mathematical formulas and symbols (such as "=" or "≥") shall be consistent. In some cases proper space around them.**

**Response:** Thanks very much for your comments. We've changed the spacing of formulas or symbols in the text to equal and marked them in red.

**4. Carefully modify the format of the reference.**

**Response：**We are based on your opinion. We have revised the reference.

**5. The study did not adequately detail the methods for processing missing data and outliers, which could potentially compromise the accuracy of the data analysis results. To enhance the reliability and scientific rigor of the study, it is recommended that the specific steps for data cleaning, as well as the statistical techniques employed to address missing data and outliers, be clearly outlined.**

**Response：**Thanks very much for your comments. Your opinion is very important to us, and we have made the following changes according to your opinion:We meticulously cleaned, screened, and organized the data in alignment with the objectives of the research. Initially, specific time periods and diseases were identified, and data were excluded for individuals who were not present in the area during their lifetime, as well as for those who resided in the area for less than five years prior to their death. We subsequently removed any missing values and cross-verified values that deviated by more than three standard deviations from the long-term mean.

**6. Although the article mentions the sensitivity analysis, it does not specify the specific content and results of the sensitivity analysis. It is recommended to add a detailed introduction of this part to increase the credibility of the conclusion.**

**Response：**We appreciate the expert advice provided. In the sensitivity analysis results of this article, we opted to present the findings from the representative and universal models due to the large number of models and factors involved. This approach further emphasizes the robustness of both the results and the model. We have included the degree of freedom selection and results for specific factors within particular models in the table located in the supplementary material. In response to expert feedback, we have revised certain formats in the sensitivity results to enhance clarity and accuracy. The sensitivity analysis results are available in the supplementary material.

**7.The paper does not specify the construction process and rationality of the analytical model. The authors are advised to specify each step of model construction in the 'Materials and Methods' section and to discuss the applicability and limitations of the selected model.**

**Response：**Thank you very much for your expert opinion. Based on your feedback, we have crafted a detailed introduction regarding the selection of the model and have introduced the applicability of the two models in turn. The content is as follows: Initially, we employ the Generalized Additive Model (GAM) to analyze nonlinear relationships within time series data, effectively fitting potential patterns and trends using various smoothing functions. Subsequently, we utilize the Distributed Lag Non-Linear Model (DLNM) to dynamically capture the temporal and dose-response effects, addressing the intricate relationships between environmental factors and health outcomes. This approach culminates in the establishment of an exposure-lag-response model. When used in conjunction, the two can fully leverage their complementary characteristics, thereby enhancing the novelty, depth, and breadth of the analysis.

**8. In the discussion of practical applications, recommendations can be more comprehensive and specific, for example, how to combine the specific work of local health departments and how to communicate and cooperate with the public to improve the effectiveness of early warning measures.**

**Response：**Thank you very much for your professional advice. Based on your suggestions, we have made several additions and modifications to the discussion section and the establishment of the early warning model, as follows: In light of the potential exposure lag response curve, cumulative relative risk results, and the effectiveness of risk early warning systems, we have thoroughly considered and discussed the establishment of an early warning system. Firstly, we recommend that the local climate environment, as well as economic and social conditions, be comprehensively evaluated. The thresholds for various influencing factors can vary significantly depending on the climate zone, and economic conditions may also influence the quality and standards of the early warning system. Secondly, given the hysteresis associated with the impacts, we propose extending the setting time for the early warning system and issuing alerts within one week following the occurrence of comprehensive exposure events to enhance population protection and prevention efforts. Furthermore, for the specific development and dissemination of the early warning system, we suggest collaboration with relevant agencies, such as the local CDC. This partnership should leverage the expertise of the CDC’s infectious disease and environmental institutes, ensuring mutual support in providing technical assistance. Subsequently, relevant outreach departments should promote the system through various media channels to ensure that the majority of residents understand, recognize, and accept this system, thereby enhancing its practical implementation. Lastly, we recommend categorizing heat wave warning levels for exposure events into three tiers: low (T2), medium (T1), and high (T3). Given the heightened risks associated with combined events, we further propose dividing pollutant warnings into three categories based on the definitions of AQI and BC concentrations: '+Low', '+Medium', and '+High'.

**9. Discuss one or more specific public health prevention strategies or interventions and how they can be integrated into the current health system.**

**Response：**Thank you for your expert advice. In response to your comments, we have made corresponding additions to the discussion section. The additions are as follows: The THI's early warning system can be integrated with the existing heat wave early warning system. The simultaneous activation of similar components in both systems can reduce the costs associated with issuing early warnings. Moreover, the THI early warning system exhibits greater sensitivity in detecting changes in the external dynamic environment and takes into account the specific characteristics of the local environment. This provides relevant health departments with more scientific and accurate foundational information for effectively addressing heat and humidity incidents.

**Reviewer#2：**

**The overall presentation of the manuscript is clear and the discussion is sufficient, but it is still recommended to modify the following to improve the quality of the manuscript:
1.The title of the article gives the illusion that it is a combination of three factors , but it is actually the interaction of THI and the other two that needs to be adjusted not to mislead the reader.**

**Response**：We sincerely appreciate your expert advice. The insights provided are both professional and relevant. In response to your comments, we have made corresponding improvements to the title. The modifications are as follows: **Exploring Temperature and Humidity Environment Combined with Air Quality Index,Black carbon,the short-term Effect of Combined Exposure on Respiratory Disease Mortality in Southwest China**

**2.The abstract needs to be rewritten: T3A3, ER with full spelling and then abbreviations.**

**Response**: Thanks very much for your comments. We have added the full name written in the article.

**3.The specific location, total population, climate and demographic characteristics of the study are missing. What is the specific area?**

**Response：**Thank you very much for your expert advice. In response to your comments, we have made several additions and modifications to the study location section. The changes are as follows: The study area is situated in the Sichuan Basin, China, specifically in the southwest region of the Chengdu Plain. It experiences a humid subtropical climate characterized by high temperatures and humidity throughout the year. Surrounded by mountains, this area faces significant pollution challenges, making it one of the most polluted regions in China. As of the end of 2023, the permanent population was recorded at 2.955 million, with men and women each representing 50.00% of the total. The demographic distribution shows that individuals aged 0-14 comprise 14.01% of the population, those aged 15-59 account for 61.26%, and the population aged 60 and above constitutes 24.73%, with individuals aged 65 and above making up 20.02%.

**4.Meteorological data and air quality data sources are downloaded or generated by yourself, to mark the reference and tell the generation process, is the data of several sites, etc., be more unclear.**

**Response**：Thank you very much for your expert opinion. We acknowledge that this was an oversight on our part, and we have made the necessary additions in response to your comments. The modifications are as follows: Both meteorological and air pollutant data were 1×1km raster data, and patient addresses were API matched to calculate individual exposure for each patient.

**5.In the statistical analysis section, FIG2 and TABLE2 should not appear and need to be modified.
wrong formula? g(X) = ols((y, df)+X)? Formula 2 dow has a coefficient y, right?**

**Response：**Thank you for your valuable feedback. We have made the necessary deletions in accordance with your suggestions. We have also simplified the formula, provided a clearer explanation of its components, and highlighted these changes in red. The modifications are as follows: Y = β0 + β1X1 + β2X2 + … + βnXn + ϵ. Y stands for the dependent variable (response variable). β0 represents the intercept, which represents the predicted value of the dependent variable Y when all independent variables X are zero. β1, β2,..., βk represent the slope coefficients, which represent the expected change in the dependent variable Y when an independent variable Xi is increased by one unit. Each coefficient represents the degree to which the independent variable affects the dependent variable. X, X1, X2, ,..., Xk represent the independent variable (explanatory variable) and are used to predict the dependent variable Y. ε represents the error term, which represents the difference between the predicted value of the model and the actual observed value.

**6.Joint meteorological events are categorical variables, binary classification? How to build a model to calculate RR should be explained in detail and attached with references.**

**Response：**Thank you for your valuable suggestions. In response to your feedback, we have made appropriate additions to the results of the exposure event, outlined as follows: The combined meteorological event variables were the continuous variables. Based on your suggestions, we have made corresponding modifications to the RR calculation section and incorporated the relevant literature. The modifications are highlighted in red.

**7.The formula for calculating RR is 10*β?? Isn't it an IQR change?**

**Response：**Thank you very much for highlighting our writing errors. We utilize the IQR change when calculating the RR, and we have made the necessary adjustments based on your feedback. The modifications are as follows:：RR=exp(IQR*β).

**8.And table 2 also not accurate, such as B1 does not contain B2? Is it an inclusive relationship?**

**Response：**Thank you for your valuable feedback. In response to your suggestions, we have enhanced the Statistical Analysis section by including further explanations regarding the source of each definition, as well as clarifying the rationale behind our classification. The revised content is as follows: The definition of exposure events is informed by the RCS exposure response curve, in conjunction with the 'GB3095-2012' standard established by the World Meteorological Organization (WMO), the National Meteorological Administration, and the China Meteorological Administration for heat wave definitions, as well as previous research. We categorize the Temperature-Humidity Index (THI) as follows: T1 (THI < 63), T2 (63 ≤ THI < 75), and T3 (THI ≥ 75). Furthermore, by integrating the BC and AQI percentiles from this study and referencing the Technical Specifications for Ambient Air Quality Assessment (HJ6632013), the Guidelines for the Preparation of Urban Heavy Air Pollution Emergency Plans, and the Sichuan Province Heavy Pollution Weather Emergency Plan (2018 Revision) - Quantitative Indicators, we redefine the BC and AQI categories as B1 (P ≥50), B2 (P ≥75), B3 (P ≥ 90), A1 (P ≥50), A2 (P ≥75), and A3 (P ≥90). Notably, A1 encompasses A2 and A3, while B1 includes B2 and B3. This study utilizes the RCS intersection value as a node, treating the combined meteorological event variable as a continuous variable, thereby fixing it within the ranges above or below different percentiles. This approach aims to investigate the relationship between THI and extreme AQI and BC, as well as to explore the impact of exposure to combined events with varying threshold ranges on respiratory mortality, ultimately refining the thresholds for early warning models.

**9.Each analysis method should be marked with related references.**

**Response:** We sincerely appreciate your expert advice, which has significantly enhanced our understanding. In response to your suggestions, we have enriched the literature review for each analysis method. The specific modifications are highlighted in red within the text.

**10.THI minimum 67 is not shown? The categorical values in Table 2 lack explanation and reason.**

**Response：**Thank you for your valuable feedback; we acknowledge this oversight in our analysis. In response to your suggestions, we have revised the RCS results plot of THI presented in Figure 1. Additionally, we have included an explanation of the classification criteria for Table 2, as per your recommendations, and have highlighted this section in red for clarity. The modifications align with your inquiry in question 8. The revised content is as follows: This study utilizes the RCS intersection value as a node, treating the combined meteorological event variable as a continuous variable, thereby fixing it within the ranges above or below different percentiles. This approach aims to investigate the relationship between THI and extreme AQI and BC, as well as to explore the impact of exposure to combined events with varying threshold ranges on respiratory mortality, ultimately refining the thresholds for early warning models.

**11.The resulting statement in Figure 2 is ER but the graph is RR? The figure also lacks confidence intervals and is incomplete.**

**Response：**We appreciate your insightful comments regarding our article. We acknowledge that there was an oversight in our initial writing. In the calculation method, we provide the formula for calculating the ER. In the explanatory text, we can utilize either the ER value, defined as (RR - 1) * 100%, or the RR for clarity. To ensure consistency with the expression used in the figure, and to enhance understanding for our readers, editors, and reviewers, we have opted to use the RR value in the explanation, which is highlighted in red in the text. To improve the clarity and intuitiveness of Figure 2, we chose not to include the confidence intervals directly in the figure. So, the results of the confidence intervals are detailed in Tables S1-S7 in the supplementary material.

**12.The asterisk in Figure 3 is redundant.**

**Response：**Thank you very much for your expert opinion. Based on your feedback, we have removed the asterisk from Figure 3.

**13.Discussion to increase how these three factors might combine? Is it possible that weather interacts with each other.**

**Response**：Thank you very much for your expert opinion. Based on your suggestions, we have expanded the discussion to include how the three factors may be combined and whether there are any interactions among the weather variables. The additional content is as follows: The calculation of the AQI primarily relies on monitoring the concentrations of various air pollutants. These pollutants typically include PM_2.5,_ PM_10_, O_3_, SO_2_, and CO. BC is a component of PM_2.5_; therefore, fluctuations in BC concentration are largely dependent on changes in PM_2.5_ concentration. However, due to the rapid changes in climate, alterations in its material properties, and shifts in its pathogenic mechanisms, an increasing number of studies are now beginning to investigate its effects on the human body separately^34^.This is one of the reasons why BC is examined independently in this study. As a combination of exposure events, BC can be studied in conjunction with other pollutants such as SO_2_, NO_2_, and O_3_ to investigate the effects of their combined exposure on the population. The environment we inhabit is not made up of a single substance or event. The occurrence of extreme temperatures, sunshine, precipitation, cyclones, and other weather phenomena frequently alters our lifestyle and surroundings. Relevant studies have demonstrated that short-term exposure to elevated levels of high temperatures, NO_2_, O_3_, and CO is significantly associated with an increased risk of respiratory mortality. Furthermore, men and the elderly are particularly susceptible to these environmental factors^35^. Du found that climate factors and air pollution have lagged and nonlinear effects on the epidemic of hand, foot, and mouth disease. Furthermore, the influence of climate factors on health may vary based on the AQI^36^. There is a connection between various weather types and air pollutants. For instance, coastal cities are more vulnerable to different patterns of atmospheric circulation and types of cyclones^37^.In the context of climate change, it is essential to focus on the bidirectional interaction between meteorological conditions and air pollutants, as well as the effects of their combined exposure on the population. This emphasis underscores the significance of our research.

34.Li Y, Zheng C, An X, Hou Q. Acute effects of black carbon on mortality in nine megacities of China, 2008-2016: a time-stratified case-crossover study. *Environ Sci Pollut Res Int*. 2022;29(38):57873-57884. doi:10.1007/s11356-022-19899-0

35.Shao M, Yu L, Xiao C, et al. Short-term effects of ambient temperature and pollutants on the mortality of respiratory diseases: A time-series analysis in Hefei, China. *Ecotoxicol Environ Saf*. 2021;215:112160. doi:10.1016/j.ecoenv.2021.112160

36.Du Z, Lawrence WR, Zhang W, Zhang D, Yu S, Hao Y. Interactions between climate factors and air pollution on daily HFMD cases: A time series study in Guangdong, China. *Sci Total Environ*. 2019;656:1358-1364. doi:10.1016/j.scitotenv.2018.11.391
